# Integrating SAXS/WAXS with Molecular Dynamics: RNA Nanoparticles as a Case Study at the Life Sciences X-ray Scattering Beamline (LIX), NSLS-II

**DOI:** 10.1063/4.0000971

**Published:** 2025-10-27

**Authors:** James Byrnes, Kriti Chopra, Kirill Afonin, Lewis A Rolband, Joanna K Krueger, Hubertus JJ van Dam, Leyla Danai, Damian Beasock

**Affiliations:** 1 Brookhaven National Laboratory, Upton, NY, USA; 2 UNC Charlotte, Charlotte, NC, USA

## Abstract

RNA nanoparticles (NANPs) hold great potential as therapeutics for gene regulation, but their structural flexibility presents challenges in understanding their immunostimulatory properties. Small-Angle X-ray Scattering (SAXS) combined with Wide-Angle X-ray Scattering (WAXS) provides crucial insight into NANP flexibility, which, when integrated with Molecular Dynamics (MD), helps elucidate the structural basis of this behavior. In this talk, I will discuss the experimental design and SAXS/WAXS data collection of NANPs at the Life Sciences X-ray Scattering beamline (LIX) at NSLS-II.

